# Clinical Outcomes of Home-Based Palliative Care: An Advance Illness Management Program

**DOI:** 10.1093/geroni/igab046.070

**Published:** 2021-12-17

**Authors:** Cara Wallace, Yit Mui Khoo

**Affiliations:** 1 Saint Louis University, St. Louis, Missouri, United States; 2 Saint Louis University, Saint Louis, Missouri, United States

## Abstract

Prior studies have reported evidence that patients with chronic medical conditions benefit from home-based palliative care to manage symptoms. The purpose of this study is to evaluate the efficacy of an advanced illness management program provided by a visiting nurse agency. This program aims to reduce the burden of illness and to manage symptoms of patients who have difficulty leaving their home due to severity of their medical conditions. Data for this study were collected from patients who received home-based palliative care from the agency. Although the program has enrolled close to 500 patients from 2016 to 2019, the analytic sample for this study was restricted to patients enrolled between 2018 – 2019 who completed both baseline and follow-up assessments for three measures: Rapid Geriatric Assessment, Integrated Palliative Care Outcome Scale-Patient Version (IPOS), and Brief Illness Perception Questionnaire (BIPQ; N=96, capturing 33.8% of eligible patients). Paired sample t-tests were used to compare the symptoms and health outcomes between baseline and follow-up assessments. Average age of the participants were 79.9 years. Results from the RGA measure showed that patients’ scores on frailty and sarcopenia were significantly lower at follow-up, indicating improvement. Comparison of scores between baseline and follow-up on the IPOS measure showed that patients experienced improvement in the following symptoms: pain, weakness, nausea, poor appetite, constipation, sore/dry mouth, drowsiness, and mobility. Findings suggest that palliative care services can effectively managed the symptoms and health outcomes of homebound chronically ill patients. Other implications include reduced emergency room visits and hospital admissions.

